# From quality to clarity: evaluating the effectiveness of online ınformation related to septic arthritis

**DOI:** 10.1186/s13018-023-04181-x

**Published:** 2023-09-15

**Authors:** Fatih Golgelioglu, Sebati Baser Canbaz

**Affiliations:** 1 Department of Orthopedics and Traumatology, Elazığ Fethi Sekin City Hospital, Doğukent Location, 23280 Elazığ, Turkey; 2 Department of Orthopedics and Traumatology, Faculty of Medicine, Erciyes University, Kayseri, Turkey

**Keywords:** Septic arthritis, Websites, Online information, Joint infection

## Abstract

**Background:**

The aim of this study was to assess the content, readability, and quality of online resources on septic arthritis, a crucial orthopedic condition necessitating immediate diagnosis and treatment to avert serious complications, with a particular focus on the relevance to individuals from the general public.

**Methods:**

Two search terms (“septic arthritis” and “joint infection”) were input into three different search engines on the Internet (Google, Yahoo, and Bing) and 60 websites were evaluated, with the top 20 results in each search engine. The websites underwent categorization based on their type, and their content and quality were assessed utilizing the DISCERN score, the Journal of the American Medical Association (JAMA) benchmark, the Global Quality Score (GQS), and the Information Value Score (IVS). The readability of the text was assessed through the utilization of the Flesch Kincaid Grade Level (FKGL) and the Flesch Reading Ease Score (FKRS). The presence or absence of the Health on Net (HON) code was evaluated on each website.

**Results:**

The DISCERN, JAMA, GQS, FKGL, and IVS scores of the academic category were found to be substantially greater when compared with the physician, medical, and commercial categories. But at the same time, academic sites had high readability scores. Websites with HON code had significantly higher average FKGL, FCRS, DISCERN, JAMA, GQS, and IVS scores than those without.

**Conclusion:**

The quality of websites giving information on septic arthritis was variable and not optimal. Although the content of the academic group was of higher quality, it could be difficult to understand. One of the key responsibilities of healthcare professionals should be to provide high quality and comprehensible information concerning joint infections on reputable academic platforms, thereby facilitating patients in attaining a fundamental level of health literacy.

## Introduction

Septic arthritis is a critical orthopedic condition requiring prompt diagnosis and treatment to prevent severe complications [1, 2]. This condition is usually characterized by a bacterial infection that affects the joints, leading to inflammation, pain, and stiffness [3]. In the general population, the annual incidence of septic arthritis is estimated to be between 2 and 10/100.000 people [4]. Septic arthritis can occur in any joint in the body, but it most commonly affects the knee, hip, and shoulder [5]. Patients at higher risk of developing this condition include those with compromised immune systems, preexisting joint disease, and recent joint surgery [6, 7]. If left untreated, septic arthritis can cause significant damage to the joint and surrounding tissues, resulting in long-term disability and chronic pain. In advanced cases, patients may require joint replacement surgery, which can be costly and associated with a long recovery time.

Patients now have increased access to information due to the widespread use of the Internet as a source of data [8, 9]. In order to learn more about the diagnosis, management, and treatment of septic arthritis, patients, families, and healthcare professionals are turning more frequently to the Internet.

The accessibility and convenience of the Internet have changed the traditional dynamic of the doctor-patient relationship. Patients today have access to an unprecedented amount of medical information and are increasingly involved in the decision-making process regarding their care [10, 11]. While this has many positive implications, it can also lead to potential pitfalls in the diagnosis and treatment of septic arthritis. In some cases, self-diagnosis or reliance on unverified information can lead to a delay in seeking proper medical attention. This delay can have serious consequences for patients with septic arthritis, as timely diagnosis and treatment are essential for good outcomes. Moreover, the Internet is rife with inaccurate and misleading information, which can negatively impact patient psychology and cause further complications [12].

Therefore, it is crucial for patients and healthcare providers to critically evaluate the reliability and quality of the information found on the Internet. This involves assessing the source of the information, seeking evidence-based materials from reputable sources, and being wary of misleading information. By taking these measures, patients and healthcare providers can make informed decisions about the management of septic arthritis, ultimately leading to better patient outcomes. Given the importance of accurate and reliable information for treating septic arthritis, there is a need to evaluate the validity and usefulness of the material available on the Internet. Limited research exists about online patient resources about septic arthritis. The purpose of this research was, thus, to assess the content, readability, and quality of online resources about septic arthritis. By addressing these issues, this study aims to enhance our understanding of the role played by online resources in supporting patient education, self-management, and decision-making regarding septic arthritis. Ultimately, it seeks to contribute to the improvement of patient outcomes by promoting the critical evaluation of online information, fostering health literacy, and empowering individuals to make informed decisions about their health care.

## Materials and methods

This investigation followed the methodology described in a recent paper by Agar et al. [13] to provide the scientific reliability. The websites were chosen using a combination of search terms that patients are most likely to use to seek information about the procedure. These search terms were “septic arthritis” and “joint infection.” As of February 2023, Google had an 80% market share, followed by Bing (15%) and Yahoo! (3%) [14]. Both search terms were inputted into three popular search engines (Google, Yahoo, and Bing), resulting in six distinct searches (2 search terms, 3 search engines). The top 10 results from each search were recorded, for a total of 60 websites. All searches were done on the same day, February 19, 2023, and all browsers' cookies were deleted before searching. Keywords and search engines separated websites. When a duplicate website was identified, the search term and engine were chosen based on which combination returned the highest-ranked (i.e., earliest) website for a given query. Video contents were also excluded from the study. Two orthopedic and traumatology specialists independently assessed each website's content and evaluation scores. Before analysis, a third orthopedic and traumatology specialist resolved any authorship or content conflicts. The websites were classified into four distinct categories, including academic, physician, medical (health-related websites), and commercial. To define the types of academic, physician, medical, and commercial resources, specific criteria were established based on multiple factors. These criteria encompassed various aspects, including content, domain, source, and publisher. Academic resources were identified as websites affiliated with recognized educational institutions, research organizations, or scholarly journals, focusing on disseminating peer-reviewed scientific knowledge. Physician resources referred to websites associated with healthcare professionals, such as individual practitioners, medical clinics, or hospitals, which provide medical information, treatment guidelines, or clinical expertise. Medical resources encompassed health-related websites that aim to educate patients, provide general medical information, or address health concerns. Commercial resources were classified as websites associated with pharmaceutical companies, medical device manufacturers, or healthcare-related businesses that may offer products, services, or advertisements.

## Methods of assessment

Using the DISCERN tool [15] for determining the reliability of written health information, all relevant websites were examined for the Journal of the American Medical Association (JAMA) benchmark, the Global Quality Score (GQS), the Flesch-Kincaid Readability Test Tool (FK), the presence or absence of the Health On the Net (HON) Foundation seal, and the information value score (IVS).

The DISCERN questionnaire assisted individuals in providing consumers with a universally accepted measure of the quality of health information. The focus of this exercise is on evaluating written materials relevant to health care. In 1998, as part of a nationwide effort in Britain, the instrument was validated to set minimum standards for the quality of written information on treatment options made available to the public by nonprofits, the National Health Service, self-help groups, the pharmaceutical sector, and other industries. The DISCERN instrument consists of 16 questions ranked on a 5-point scale. For each criterion, a score of 1 indicates that it is not met at all, 2–4 indicates that it is met to some extent, and a score of 5 indicates that it is met completely. As the response options for each question range from 1 to 5, the lowest and highest total scores for this instrument are 6 and 80, respectively. Websites were ranked as “excellent” (63–80), “good” (52–61), “medium” (39–50), “poor” (28–38), or “very poor” (27) based on their aggregated scores [15].

Four factors are used in JAMA's benchmark evaluation of the quality of online content (authorship, attribution, description, and currency) [16]. Authorship requires that a website provide the names, affiliations, and links of all authors and collaborators. References and materials used in creating content must be acknowledged, and copyright information must be included. Website ownership information, including any and all commercial, financial, and possible conflicts of interest, should be made available to the public. Last but not least, currency guarantees that the website's material is dated at the time of the original upload and future revisions. Scores ranged from 0 (the lowest quality) to 4 (the highest quality) based on how many of the criteria were met.

In addition, the Global Quality Score (GQS), which is a statistic that ranks the overall quality of the websites and consists of a scale of 5 points, was determined for each website that was included in our analysis [17]. The ratings assigned to the website reflected both the quality of the information it provided and the potential advantages it offered to patients.

The Flesch-Kincaid Grade Level (FKGL) assessment is utilized for determining the educational proficiency necessary for comprehending a given text. The FKGL score ranges from fifth grade to college graduate level, with 5 being the minimum level and 12 being the maximum. The article's readability was measured with the Flesch-Kincaid Reading Ease (FKRS) test. The score informs the reader of the approximate education level needed to understand a given text. A number between zero and one hundred was assigned to a piece of writing to signify how easily it could be understood. Scores at or near 100 indicated the document was easy to read, but scores at or near zero indicated the document was difficult to comprehend. To acquire FK scores, the text of each website was copied to a Microsoft Word (Redmond, Washington) document, a method utilized in prior studies [18, 19].

We investigated whether or not websites were in compliance with the Health on the Net Code (HON code). The recommendations provided by the HON Foundation are now the credibility standard that is utilized most frequently for online medical information. In 1995, a non-profit organization based in Switzerland established the foundation with the goal of elevating the standard of health information provided on the Internet. The foundation offers a code of conduct for websites that acknowledge its ideals and adhere to its standards; compliance with the code is audited in a random way to verify that it is being respected [20].

We have also developed our own scoring system based on the information value of the data presented on the sites to make our assessment similar to previous studies [21, 22]. Information value score (IVS) is a 100-point rating scale comprised of four major factors: disease summary (maximum 40 points), diagnosis and treatment (maximum 20 points), pathogeneses and risk factors (maximum 20 points), and complications (maximum 20 points). The summary of the disease mentions the emergency of the disease at 20 points: Five points for pain, swelling, redness, and limited mobility for a total of 40 points. The treatment and diagnosis mention five points for joint fluid analysis, blood laboratory analysis, joint debridement, and antibiotics, for a total of 20 points. Pathogenesis and risk factors were accepted as 20 points in total. Five points each were received when any of the following pathogens were mentioned: bacteria and other pathogens. Each of the following risk variables received two points for a total of 10 points: suspected sexual activity, immunosuppressed disorders, tick bite, recent joint surgery, and joint trauma. Each of the following complications received five points for a total of 20: cartilage damage, joint dysfunction, osteomyelitis, and septicemia.

### Statistics

The study findings were analyzed using IBM SPSS Statistics 22, which was provided by SPSS IBM in Turkey. The Kolmogorov–Smirnov and Shapiro–Wilks tests were used to evaluate the suitability of the normal distribution parameters. In addition to utilizing descriptive statistical methods such as mean, standard deviation, median, and frequency to compare categories, the Kruskal–Wallis test was also employed. The group that was responsible for the discrepancy was determined using Dunn's test. Non-normally distributed characteristics were compared using the Mann–Whitney U test. The Spearman's rho correlation analysis was performed to analyze the correlations between non-normally distributed factors. To establish the degrees of agreement among observers, the intra-class correlation coefficient, as well as its minimum and maximum values, was determined. The significance threshold was established at *p* < 0.05.

## Results

**Fig. 1:**
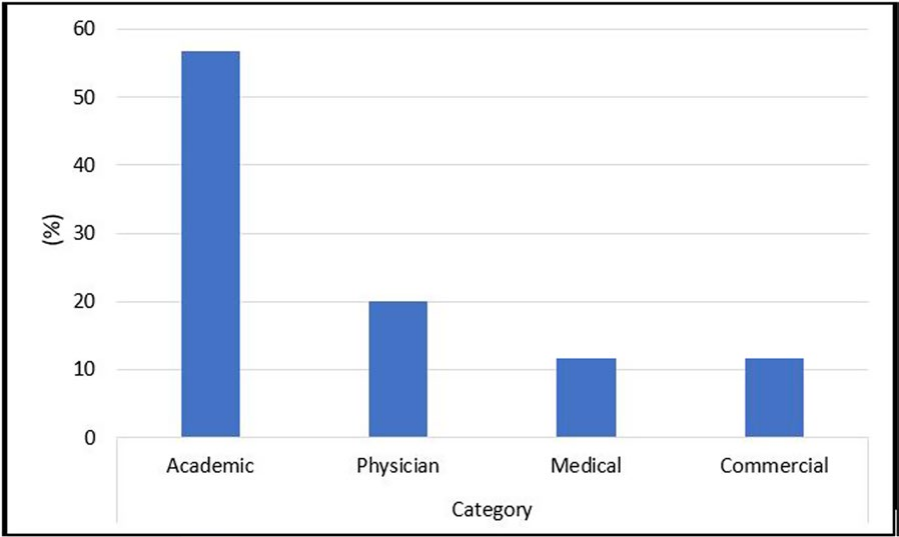
Website distribution according to sources

Initially, 60 websites were categorized based on their sources: 56.7% were academic, 20% were physician, 11.7% were medical, and 11.7% were commercial (Fig. 1).

The mean DISCERN score was 55.02 ± 18.62, the mean JAMA benchmark score was 3.18 ± 1.02, and also the mean FKRS and FKGL scores were 37.6 ± 18.75 and 10.06 ± 2.06, respectively (Table 1).

**Table 1:** Minimum, maximum, mean, standard deviation and median values of scores

	Min–Max	Mean ± SD	Median
DISCERN	20.5–78.5	55.02 ± 18.62	61
GQS	1–5	3.93 ± 1.36	4.5
JAMA	1–4	3.18 ± 1.02	3.5
FKRS	10–84	37.6 ± 18.75	35.5
FKGL	5.4–12	10.06 ± 2.06	10.8
IVS	29–100	83.18 ± 19.8	93

The study revealed that academic websites exhibited significantly higher average scores across multiple evaluation metrics compared to physician, medical, and commercial websites. Specifically, academic websites demonstrated superior performance in DISCERN (69.51 ± 7.19), GQS (4.85 ± 0.31), JAMA (3.93 ± 0.18), FKGL (11.35 ± 0.9), and IVS (96.47 ± 4.06). Conversely, commercial websites exhibited the lowest average scores in DISCERN (24.5 ± 3.52), GQS (1 ± 0), JAMA (1 ± 0), FKGL (6.51 ± 1.45), and IVS (47.14 ± 16.71). Further post hoc analyses were conducted to identify the categories that contributed significantly to these findings (Table 2), and it was found that the academic category consistently outperformed the physician, medical, and commercial categories in terms of DISCERN, JAMA, GQS, FKGL, and IVS scores.

**Table 2:** Scores from each category

Categories	DISCERN	GQS	JAMA	FKRS	FKGL	IVS
	Mean ± SD (median)	Mean ± SD (median)	Mean ± SD (median)	Mean ± SD (median)	Mean ± SD (median)	Mean ± SD (median)
Academic	69.51 ± 7.19 (70.8)	4.85 ± 0.3 1(5)	3.93 ± 0.18 (4)	25.26 ± 11.13 (22.5)	11.35 ± 0.9 (12)	96.47 ± 4.06 (98)
Physician	43.75 ± 5.84 (44.3)	3.75 ± 0.45 (4)	2.88 ± 0.23 (3)	48.08 ± 10.29 (50)	8.98 ± 1.45 (8.8)	80.42 ± 9.14 (79.5)
Medical	34.43 ± 5.05 (34.5)	2.64 ± 0.69 (2.5)	2.21 ± 0.27 (2)	49.86 ± 11.14 (49)	9.19 ± 1.98 (9.5)	59.43 ± 11.97 (56)
Commercial	24.5 ± 3.52 (24)	1 ± 0 (1)	1 ± 0 (1)	67.29 ± 13.16 (70)	6.51 ± 1.45 (6)	47.14 ± 16.71 (51)
*p*	0.000*	0.000*	0.000*	0.000*	0.000*	0.000*

Kruskal Wallis Test

*p < 0.05

There was a strong positive correlation between the first and second observers' DISCERN, JAMA, and GQS scores(*p* < 0.05). The degree of correlation among the two observers was 0.988 for the DISCERN score, 0.915 for the JAMA score, and 0.952 for the GQS score (Table 3).

**Table 3:** Level of inter-observer JAMA, DISCERN, and GQS scores' compatibility

	ICC	95% CI		*p*
		Lower	Upper	
DISCERN score	0.988	0.979	0.993	0.000*
JAMA score	0.915	0.862	0.948	0.000*
GQS score	0.952	0.920	0.971	0.000*

ICC: Intraclass correlation coefficient

*p < 0.05

The DISCERN and JAMA scores were found to have a strong positive correlation of 0.877 and a relationship (*p* < 0.05). There were also strong positive correlation found between the DISCERN and IVS scores at the level of 0.849, between the JAMA and IVS scores at the level of 0.865, and between the GQS and IVS scores at the level of 0.818 (*p* < 0.05) (Table 4).

**Table 4:** Correlation between scores

	DISCERN	GQS	JAMA	FKRS	FKGL
GQS					
*r*	0.877	–	–	–	–
*p*	0.000*	–	–	–	–
JAMA					
*r*	0.891	0.975	–	–	–
*p*	0.000*	0.000*	–	–	–
FKRS					
*r*	− 0.834	− 0.856	− 0.830	–	–
*p*	0.000*	0.000*	0.000*	–	–
FKGL					
*r*	0.806	0.855	0.830	− 0.956	–
*p*	0.000*	0.000*	0.000*	0.000*	–
IVS					
*r*	0.849	0.818	0.865	− 0.724	0.702
*p*	0.000*	0.000*	0.000*	0.000*	0.000*

Spearman rho correlation analysis

*p < 0.05

Moreover, a HON code was present on 76.7% of the websites. The websites that had a HON code had significantly higher DISCERN score values than the websites that did not have a HON code (*p* < 0.05). In addition, the JAMA, GQS, FKGL, and IVS scores of websites with a HON code were significantly higher compared to those that did not have one (*p* < 0.05). However, the websites that had a HON code had signigiciantly lower FKRS score values than the websites that did not have a HON code (*p* < 0.05) (Table 5).

**Table 5:** Evaluation of scores dependent on the presence of the HON code

	HON code		*p*
	Absent	Present	
	Mean ± SD (median)	Mean ± SD (median)	
DISCERN	41.43 ± 21.83 (32)	59.15 ± 15.56 (63)	0.005*
GQS	2.68 ± 1.74 (2.3)	4.3 ± 0.96 (5)	0.002*
JAMA	2.25 ± 1.31 (2)	3.46 ± 0.73 (4)	0.002*
FKRS	49.29 ± 21.02 (46)	34.04 ± 16.67 (34.5)	0.024*
FKGL	8.71 ± 2.52 (9.8)	10.47 ± 1.73 (11.1)	0.010*
IVS	67 ± 23.34 (66.5)	88.11 ± 15.82 (95)	0.002*

## Discussion

The most important finding of the study was that the quality of the websites providing information on septic arthritis was variable and suboptimal. Academic websites contain higher-quality information than others, but they demand a high level of expertise to be easily read, which is the second most important finding of the current study. The information available to patients online should be easily readable, reliable and of high quality because the Internet is both an information resource and a possible health education tool that can be used for the management of diseases. The patient and his or her physician need to work together to treat this disease effectively and promptly, which is necessary to avoid complications [23]. In addition, several studies have demonstrated that patient education and appropriate information increase treatment outcomes [24, 25]. This is the first systematic evaluation of the quality, readability, and content of common websites addressing septic arthritis. Many research studies have shown that the quality of Internet-based information for many medical conditions is low or non-optimal quality [19, 26, 27].

Despite the low or non-optimal quality of the information available online, patients keep depending on online resources. In this condition, the patient's resistance to the doctor's advice and the collaborative decision-making process between doctors and self-trained patients can both suffer when patients have access to the Internet. Depending on the situation, there may be delays in the treatment of septic arthritis patients. Therefore, it is crucial to review Internet resources and assist patients in locating high-quality, complete material and easily accessible websites, as low-quality information may negatively impact the relationship between patients and their doctors.

It is even possible for anyone to construct a web site without having the appropriate experience or education. When looked at from the point of view of the patient, it is challenging to evaluate what information is reliable and accurate. Managing patients who read websites offering inaccurate or low-quality information is one of the most serious difficulties facing medicine today. This might change the dynamic between a patient and doctor since patients may have different expectations for diagnosis and treatment. A physician must be aware of the information available to patients and determine what online research other physicians are conducting on a certain issue today.

In this study, the academic group's scores on DISCERN, JAMA, GQS, and IVS were all found to be considerably higher than those of the other categories. Consistent with previous studies [10, 28], we determined that information in the academic field was of the highest quality and most relevant. Some research, in contrast, demonstrated no relationship between groups and their quality scores [13]. These results demonstrate that the quality and content of the material available on the Internet, including academic studies, may be variable.

The average DISCERN score for the sample was 55.02 ± 18.62 (1–80). This conclusion is consistent with other research that have demonstrated that the quality of information provided on websites is moderate [28, 29]. Key et al., on the other hand, reported low quality scores [30]. This may be because the rate of academic sites in our investigation is greater than the rate in their study. Moreover, the websites belonging to the academic group exhibited higher DISCERN scores in comparison with the other websites. This suggests that academic websites offer information that is more reliable and of better quality when it concerns DISCERN scoring. The publications were evaluated using the DISCERN overall score, which yielded a moderate rating. This suggests that the websites are a valuable source of information but could be further improved with additional information.

**Fig. 2:**
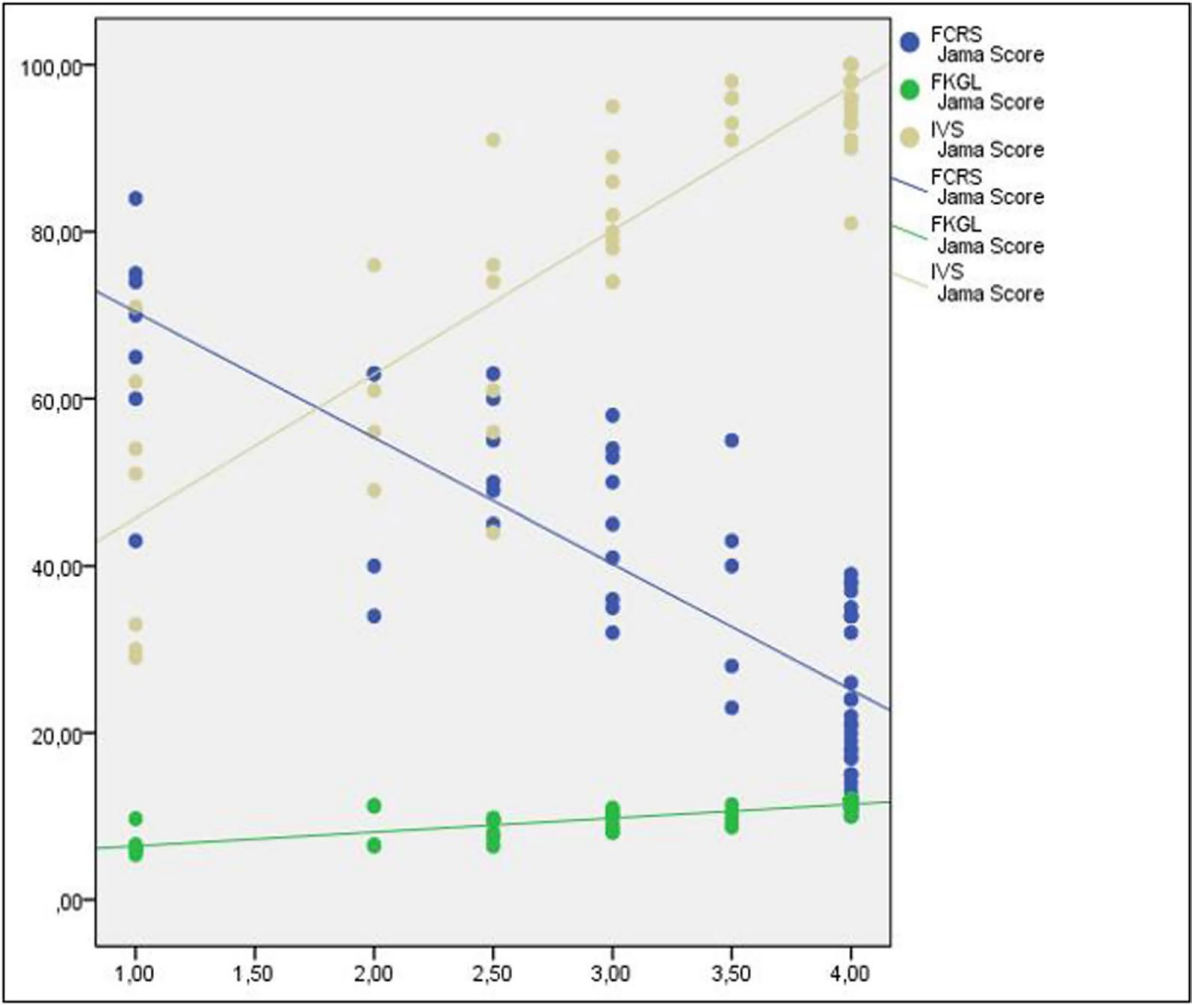
Relationship of JAMA score to other scores

The average score on the JAMA benchmark score was 3.18 ± 1.02 (0–4), which is a relatively good result. The results of this study were like those of other studies [26, 29]. Including the names and qualifications of authors is a crucial aspect of improving the credibility of a website and instilling confidence in patients. It is advisable for both patients and healthcare professionals to exercise a degree of caution when considering online information, particularly in cases where the authorship of the information is unclear and the timeliness of the information is uncertain. Although these scores are low in some other studies, we can explain this by having more academic writing in our study [10, 19]. We believe the explanation for the low JAMA scores among non-academic groups is that the majority of websites lack citations or sources. In addition, we observed a positive correlation between the JAMA benchmark criteria and the FKGL and IVS scores (*p* < 0.05) (Fig. 2).

The inference that can be drawn from this correlation suggests that websites that include authorship information and attributes are indicative of higher-quality content.

The average IVS was 83.18 ± 19.8. This content score was consistent with previous studies [31, 32]. Academic content with high IVS scores was likely written by experts on the subject. Nevertheless, not all studies have shown the same results [33, 34]. These are relatively old studies, and there may have been advancements in Internet information since then. In addition, we created this scoring system ourselves, so the results may be different when compared to previous studies.

Results of this study indicated that the average scores on the FKGL and FKRS were 10.06 ± 2.06 and 37.6 ± 18.75. We found that web pages that provided information about septic arthritis had similar readability ratings to those that examined arthroplasty, the foot and ankle, the hand, cancer, and the spine [35–38]. According to these findings, the FKRS score was at the “difficult to read” level, while the FKGL score was almost 4 degrees above the sixth-grade reading level indicated by the American Medical Association (AMA) and the National Institutes of Health (NIH). This indicates that a patient seeking information on septic arthritis online would benefit from having English language skills at or above the high school level. This is problematic because it makes it more difficult for many individuals to access the information they need about septic arthritis online.

We also discovered a positive, statistically significant correlation between GQS and IVS across all sources we looked at. This was notably true for FKGL, DISCERN, and JAMA. In the case of septic arthritis, this demonstrates a causal relationship between text readability and quality.

The majority of websites (76.7%) in this survey had a HON code. Websites with a HON code had significantly higher DISCERN, JAMA, GQS, and IVS evaluation score values compared to those without a HON code (*p* < 0.05). The findings of our research demonstrate that the HON code certification is a dependable indicator for identifying websites with superior quality and content scores in the context of Internet searches related to septic arthritis. These results are consistent with previous research examining the caliber of various healthcare subjects [39–41]. The aforementioned information is reassuring, and in our capacity as medical professionals, we can advise our patients to seek out this quality assurance marker during their online searches. However, while our study identified that websites with the HON code had significantly higher DISCERN, JAMA, GQS, and IVS scores, it is notable that these sites exhibited lower FKRS scores. This apparent contradiction could be attributed to several factors that deserve exploration. One possible explanation is that websites adhering to the HON code might feel compelled to maintain a certain level of technicality to uphold their credibility, inadvertently resulting in content that is difficult for laypeople to grasp. This trade-off between maintaining clinical accuracy and ensuring readability could contribute to the lower FKRS scores observed in HON-certified websites. Therefore, while the HON code contributes to the trustworthiness of the information, it might not necessarily address the challenge of making the content comprehensible to a wider audience.

Despite having high-quality content, academic websites have the highest degree of reading difficulty among all websites examined. Academic groups' readability scores in the current investigation showed a consistent trend of source material being pitched at a reading level far beyond what patients could easily access. Among the possible consequences of this condition is that researchers who do not comprehend the academic group's content may turn to websites with lower-quality content.

Although our findings highlight gaps in online patient education resources about septic arthritis, they also present an opportunity for improvement. The Agency for Healthcare Research and Quality (AHRQ) has published recommendations to improve health literacy and recommends taking "universal precautions" when disseminating health information. This method requires the healthcare provider to presume that everyone will have difficulty with comprehension and communicate in a manner that is universally understood [42]. Several institutions have published guidelines on how to accomplish this, including the use of simple words and phrases, careful language at lower reading level, consistent terminology, and the avoidance of excessive medical jargon [43].

To enhance the quality and accessibility of patient education resources, it is crucial for healthcare providers to actively engage in guiding patients towards reliable and easily readable websites. One effective approach is the implementation of search engine optimization (SEO) strategies [44] by academic groups and healthcare organizations that aim to provide accurate and trustworthy information to patients at appropriate readability levels. By optimizing website content and structure, utilizing clear and concise language, and employing user-friendly interfaces, these organizations can ensure that their resources rank higher in search engine results and are more easily discovered by patients seeking information on specific medical conditions. Furthermore, healthcare providers can play an active role by compiling a list of reputable websites and resources, which they can provide to patients during consultations or make available on their personal websites. This proactive approach not only empowers patients with reliable information but also fosters a stronger patient-physician dynamic, reducing the likelihood of patients relying on inaccurate or misleading information about their conditions. By embracing these strategies, healthcare providers can contribute to an improved online landscape for patient education where accurate and easily accessible resources are readily available to those in need.

Healthcare providers should actively guide patients during consultations, recommending reputable online resources and emphasizing the importance of verifying information. Encouraging patients to question sources fosters skepticism and empowers them to make informed decisions. Healthcare providers must stay updated on digital health literacy through ongoing education. Training should focus on critical appraisal of online information and evidence-based practice in the digital age.

Future studies should assess the impact of patient education interventions, explore the correlation between patients' digital health literacy and comprehension, and investigate the role of social media in shaping patient understanding of septic arthritis.

## Limitations

This study has a number of limitations. Due to the frequent updating of Internet resources, the results of our study may have changed since the initial search. After the first page of search engine results, the number of websites that a user visits during a search decreases significantly [45]. Additionally, our research only looked at written material found online, but patients may also use audio-visual content to learn about their conditions; this aspect was not evaluated. Due to the dynamic nature of the Internet, search engines and their respective ranking algorithms may alter the results or order of results frequently. Search results may still differ from person to person even after we remove cookies. Even more so, the quality of information on websites other than the three most popular search engines was not evaluated in this study. The present research employs online data, thereby rendering it susceptible to the dynamic character of the Internet. The rapid creation or updating of websites can have an impact on their ranking in search engine query results. Consequently, the websites that were analyzed may undergo rapid changes. To the best of our knowledge, this is the first study on online patient information for septic arthritis to appear in the literature.

## Conclusion

We determined that, like the quality of previous publications on orthopedic conditions, the websites providing information on septic arthritis were variable. Some online resources, particularly academic ones, have content of higher quality than others, but they are difficult to read for the general public. This makes it difficult for patients to find the information they are looking for about septic arthritis and can lead them to the inaccurate websites. With advancements in technology and the growing popularity of the Internet as a source of health information, physicians should refer patients to reliable websites and encourage the creation of content that is beneficial to patients and presented in a language and reading level they can easily read.

## Data Availability

The datasets analyzed during the current study are available from the corresponding author on reasonable request.
